# Is prehospital use of active external warming dangerous for patients with accidental hypothermia: a systematic review

**DOI:** 10.1186/s13049-020-00773-2

**Published:** 2020-08-10

**Authors:** Sigurd Mydske, Øyvind Thomassen

**Affiliations:** 1 Department of Anaesthesia and Intensive Care, Haukeland University Hospital, Bergen, Norway; 2 Mountain Medicine Research Group, University of Bergen, Bergen, Norway; 3 Department of Research and Development, Norwegian Air Ambulance Foundation, Oslo, Norway

**Keywords:** Accidental hypothermia, Active external rewarming, Prehospital, Emergency medicine, Systematic review

## Abstract

**Background:**

Optimal prehospital management and treatment of patients with accidental hypothermia is a matter of frequent debate, with controversies usually revolving around the subject of rewarming. The rule of thumb in primary emergency care and first aid for patients with accidental hypothermia has traditionally been to be refrain from prehospital active rewarming and to focus on preventing further heat loss. The potential danger of active external rewarming in a prehospital setting has previously been generally accepted among the emergency medicine community based on a fear of potential complications, such as “afterdrop”, “rewarming syndrome”, and “circum-rescue collapse”. This has led to a reluctancy from health care providers to provide patients with active external rewarming outside the hospital. Different theories and hypotheses exist for these physiological phenomena, but the scientific evidence is limited. The research question is whether the prehospital use of active external rewarming is dangerous for patients with accidental hypothermia. This systematic review intends to describe the acute unfavourable adverse effects of active external rewarming on patients with accidental hypothermia.

**Methods:**

A literature search of the Cochrane Library, MEDLINE, EMBASE, the Cumulative Index to Nursing and Allied Health Literature (CINAHL], and SveMed+ was carried out, and all articles were screened for eligibility. All article formats were included.

**Results:**

Two thousand three hundred two articles were screened, and eight articles met our search criteria. Three articles were case reports or case series, one was a prospective study, two were retrospective studies, one article was a literature review, and one article was a war report from the Napoleonic Wars.

**Conclusions:**

One of the main findings in this article was the poor scientific quality and the low number of articles meeting our inclusion criteria. When conducting this review, we found no scientific evidence of acceptable quality to prove that the use of active external rewarming is dangerous for patients with accidental hypothermia in a prehospital setting. We found several articles claiming that active external rewarming is dangerous, but most of them do not cite references or provide evidence.

## Background

Accidental hypothermia (AH] is defined as an involuntary drop in core body temperature to below 35 °C [1]. The golden standard for rewarming in severe hypothermia is the use of extracorporeal techniques, but this technology is rarely accessible outside of major trauma centres. Therefore, prehospital warming modalities are usually limited to passive external rewarming (PER) and active external rewarming (AER) techniques [2].

Hypothermia has a multitude of complications, including cardiac arrhythmia, pulmonary oedema, renal failure, hypotension, coagulopathy, and neurological pathology [1, 3–5]. Distinguishing between complications from active rewarming and the pathophysiological response to rewarming may sometimes be impossible. Hypothermia is an independent risk factor for increased mortality and morbidity in injured patients, and is also potentially fatal without any accompanying conditions [6, 7]. Given the serious consequences of being cold, any advice to abstain from warming patients should be well-documented, and the adverse effects of warming should outweigh those of being hypothermic.

The aim of this review is to identify and describe all scientific reports describing clinical complications or adverse effects occurring during rewarming of patients with AH using AER. We did not aim to report the effect of AER or the consequences of AER on core temperature afterdrop. Neither did we intend to speculate about the danger of afterdrop in patients with AH, nor to describe clinical complications of hypothermia, only the complications directly related to the use of AER. In this review, we define “complication” as any clinical haemodynamic or respiratory deterioration occurring ≤1 h after the application of AER. We decided to use this timeframe because the suspected dangerous aetiologies (i.e., vasodilatation or increased afterdrop) should present with symptoms within 1 h if they have any clinical consequences.

Therefore, asymptomatic increased afterdrop was not considered a complication. Late adverse effects of hypothermia also were not considered complications, as they may be considered consequences of hypothermia itself and not necessarily an effect of AER.

## Methods

The research protocol, including the search strategies, is available online [8]. We used the PRISMA checklist as a framework for conducting this review [9].

### Search strategies

A literature search of the Cochrane Library, MEDLINE, EMBASE, the Cumulative Index to Nursing and Allied Health Literature (CINAHL), and SveMed+ was carried out on 3 August 2018. An update search was conducted on 25 June 2020.

We used subject headings when applicable, with the addition of free-text search terms and terminology to complete the search strategy. We decided to be very liberal with both our search criteria and in the selection of included article formats. This was necessary in order to obtain all of the necessary articles for the review because of the inconsistent use of terminology in scientific literature.

### Inclusion and exclusion criteria

We used the PICO framework for focused clinical questions [10].

Population: Patients with AH.

Intervention: Patients with AH who received AER, using non-invasive or minimally invasive techniques only.

Comparison: Ideally, studies in which the intervention was compared to a control group. Outcome: Unfavourable outcome including rewarming syndrome, rewarming shock, rescue collapse, or any other negative physiological response to the intervention (i.e., cardiac arrhythmia, pulmonary oedema, renal failure, hypotension, coagulopathy, neurological pathology, or other complication).

Our search was not limited with regard to publication date, as the origin of the hypothesis regarding the resistance to AER in the prehospital setting has been relevant for centuries. There was no restriction on patient age, but we chose to exclude studies concerning hypothermia in preterm neonates, as these articles describe an intrahospital setting. We excluded articles regarding therapeutic hypothermia and invasive treatment. We also excluded animal studies.

For obvious ethical reasons, randomized controlled trials are lacking. Therefore, current guidelines and practice rely heavily on observations, case reports, and the clinical experience. Consequently, all such articles must be eligible for review and critical consideration. All languages were considered and, if necessary, translated.

### Study selection

After the searches were completed, the authors (SM and OT) conducted a double-blind title screening of 10% of the articles using Rayyan QCRI Systematic Review software (Qatar Computing Research Institute, Hamad Bin Khalifa University) [11] in order to ensure mutual understanding regarding the studies eligible for inclusion. From this, we calculated a Cohens Kappa Coefficient (휅) of 0.78 which indicate a moderate-to-strong interrater reliability [12]. We deemed this acceptable due to the fact that the reviewer who would be conducting the initial title-screening (SM) consistently included studies the second reviewer (OT) chose to exclude. Therefore, there would be minimal risk of falsely excluding relevant articles.

**Fig. 1:**
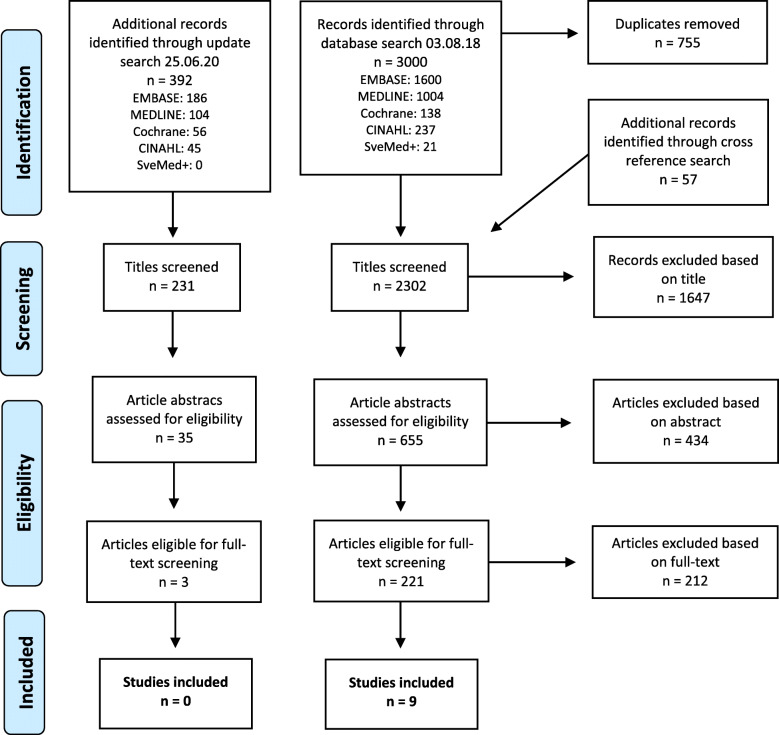
The PRISMA flow diagram showing the process of eligibility screening in our review

SM then screened the rest of the titles and excluded those that were obviously ineligible. After this, both reviewers screened the abstracts to determine eligibility using Rayyan QCRI in a double-blind manner. Finally, we conducted a double-blind full-text selection. If there was a discrepancy between the reviewers, the article was included in the next selection step (Fig. 1).

### Data synthesis and stratification

Due to the lack of randomized controlled trials and other high-quality scientific literature regarding this subject, a large number of article formats were eligible for inclusion.

### Risk of bias assessment

Evaluating the risk of bias proved to be difficult given the variety of included studies. Risk of bias was assessed using the STROBE checklist [13] for the observational studies and the CARE checklist was applied to the case reports [14]. We were unable to find applicable validated tools for the remaining article formats.

## Results

Eight articles [15–22] met our inclusion criteria for this review: Three were case reports or case series, one was a prospective study, two were retrospective studies, one article was a literature review, and one article was a war report from the Napoleonic Wars (Table 1).

**Table 1:** A summary of the relevant findings in the selected articles included in our review

Article ID	Aetiology	Temp.	Case description	AER Intervention	Complication	Quality score
Coopwood et al. 1974 [15]	Outdoor exposure, overnight	25 °C, rec	Male, 70 y; responsive to pain, unobtainable BP	Electric blanket	Initial increase in BP and T_rec_, then sudden drop in T_rec_ and BP - > VF - > †	17 / 30 CARE
Strapazzon et al. 2012 [16]	Avalanche burial, 2 h	25 °C, tymp	Male, 42 y; GCS 10, breathing, palpable radial	Forced air warming	Atrial fibrillation Pulmonary oedema Hypotension Hypoglycaemia	21 / 30 CARE
Emslie-Smith et al. 1958 [17]	Outdoor exposure, unknown duration	33 °C, rec	Female, 64 y; stupor, breathing, hypothyroidism	Electric blanket	Coma, hypotension Bronchopneumonia Death	14 / 30 CARE
**Article ID**	**Mean age**	**Mean temp**	**AER modality**	**Number of patients** **AER (Mortality in %)**	**Number of patients PER** **(Mortality in %)**	**Quality score**
Duguid et al. 1961 [18]	75.3 years	26.5 °C, rec	Not specified	6 (100%)	17 (58.8%)	8 / 22 STROBE
**Article ID**	**Number of subjects** **(# treated with AER)**	**Temp**	**AER Modality**	**AER mortality** **(PER Mortality)**	**Comments**	**Quality score**
Fruehan, 1960 [19]	8 (4)	Mean: 24.4 °C, rec^a^	Not explicitly stated	100% (75%)	4 pt. treated with some form of AER, all died; 4 pt. treated with PER, 1 survived	12 / 22 STROBE
O’Keeffe 1973 [20]	62 (1)	Below 30 °C	Immersion in hot bath	100% (9.8%)	1 pt. treated with rapid rewarming by immersion, cardiac arrest immediately after rewarming	13 / 22 STORBE
**Article ID**	**Timespan of data collection**	**Overall mortality**	**Number of cases** **(AER, PER, Internal)**	**Mortality of patients treated with AER**	**Mortality of patients treated with PER**	**Quality score**
Gregory et al. 1973 [21]	1951–1972	48.8%	201 (73, 121, 7)	60.3%	44.6%	–
**Article ID**	**Description**					
Moricheau-Beaupré, 1826 [22]	The writings of Napoleon’s regimental surgeon from the Russian campaign in 1812: “The like holds of general as of local asphyxia; we must not, in avoiding the danger from cold, transport the body into a heated place, or immediately apply to it warm substances; too strong reaction might exhaust the remaining vitality; the dilatation of the tissues and rapid expansion of the forces towards the surface, owing to sudden transition from cold and condensed to warm and rarefied air, causing shooting pains, dyspnoea, suffocation, and death.”					

^a^Endobronchial in one case

### Complications following AER

The most common complications reported from AER were a drop in blood pressure [15–17], dangerous cardiac arrythmias [15, 16] and reduced level of consciousness [15, 17], sometimes resulting in death. Four studies reported an increased mortality rate in patients receiving AER compared to patients receiving PER [18–21].

### Modality of AER with unfavourable outcome

In two of the studies reporting unfavourable outcome from AER an electric blanket was used [15, 17]. One study reports hot water immersion [20] and one reports the use of forced air warming. Three of our studies does not explicitly report the modality of AER used in the reported cases [18, 19, 21].

### Main findings

The article from the Napoleonic wars [22] concludes that a rapid rewarming is dangerous, and one should adopt a slower approach to rewarming, which is partially supported by Fruehan [19]. Gregory argues that a passive rewarming approach seems to be safer [21]. In the prospective study by Duguid, she reports that a total of 6 patients was treated with AER, all of whom promptly died [18]. Treatment with AER was deemed dangerous and was discontinued. Coopwood and Strapazzon recommends the use of AER but advises caution during treatment due to risk of potentially fatal complications [15, 16].

### Study quality

As presented in the table, the overall quality of the studies included in this review was low. The lack of high-quality research is due, at least in part, to the obvious ethical and practical challenges that make it difficult to conduct proper clinical studies on this subject.

The included cohort studies are not detailed enough to attribute the less favourable outcome of the patients in the AER group to AER itself; crucial information is missing to draw that conclusion [18–20]. However, these articles are frequently cited as the source of the dangers of AER in the medical literature. The studies are very old and have crucial methodical flaws, as indicated by their low STROBE score.

The case reports also have methodical weaknesses. The report from Emslie-Smith is a case series, but only one of the cases met our inclusion criteria [17]. The report from Strapazzon is of good quality, but it is difficult to assess whether the complications occur because of the hypothermia or the AER [16].

## Discussion

### Complications following AER and pathophysiology

Several different physiological theories have been proposed to explain why AER is dangerous, and most of them argue that AER affects afterdrop, a thermodynamic phenomenon. As dictated by the second law of thermodynamics, heat will flow from hot areas to cold areas [23]. In a patient with rapid onset AH, a significant thermal gradient is present between the core and the skin and subcutaneous tissues [24]. As the patient is removed from the cold environment, the thermal energy of the body will seek equilibrium. Heat will travel from the relatively warm core out to the colder, peripheral areas of the body, which results in a drop in the core temperature [25, 26].

A number of theories advocate the physiological dangers of AER, and the most prevalent theory argues that AER will accelerate afterdrop by alleviating peripheral vasoconstriction. Some scientists argue that the flow of blood from the relatively warm core and out through the colder peripheral tissues will increase. They believe this relatively cold blood will return from the extremities and cool the core at a faster rate, and a thermodynamic equilibrium will be instated more rapidly than without the circulatory (convective) component [26–28].

Given that the patient is properly isolated and removed from the cold environment, the total thermal energy will not decrease, and the nadir temperature will not be lower if thermal energy is supplied in the form of AER than with passive insulation alone [27]. Therefore, if this theory that AER is dangerous for patients with AH is true, it has to be due to the acceleration of the afterdrop and not an increase of the afterdrop. Both a convective and conductive mechanism is considered relevant, but the exact mechanism is not yet completely understood [27, 29].

### Main findings

Ever since the Napoleonic Wars there has been significant controversy regarding AER, with several authors discouraging this practice. Lankford wrote a very interesting article on the history of cold-related injuries [30], and James Currie described the phenomenon of afterdrop for the first time [31]. In more recent history, the infamous Dachau experiments conducted by Nazi doctor Sigmund Rascher during the second world war formed the basis for a new understanding of immersion hypothermia. The report of US Major Leo Alexander contained and preserved the results from these atrocities [32]. However, the results and conclusions drawn from these experiments are heavily flawed. A review of the Nazi experiments revealed *“critical shortcomings in scientific content and credibility. The project was conducted without an orderly experimental protocol, with inadequate methods, and an erratic execution. The report is riddled with inconsistencies. There is also evidence of data falsification and suggestions of fabrication. Many conclusions are not supported by the facts presented*” [33]. Despite the overwhelming criticism of the Dachau experiments, it may seem as though some of the conclusions survived. The Nazi scientists observed a continuous drop in core temperature, and they postulated that this drop in temperature may be responsible for dangerous cardiac arrhythmias.

Since then, many scientific papers have warned about the danger of AER, most of them without citing references [34–45]. This may indicate that this theory was sufficiently established as an undisputed truth, even though no scientific evidence supports this.

The articles included in this review, and others that did not meet our specific criteria, report a fairly high mortality rate in patients with AH. It may seem as though the vast majority of deaths occur long after treatment with AER is initiated, or even after the patient is successfully rewarmed [4, 46]. We think that, if the hypothesis of a sudden cardiovascular collapse after AER is true, then the complications should occur fairly soon after the treatment is initiated. It is unreasonable to attribute all of the late deaths from hypothermia to the use of AER, as these deaths are more likely to be a consequence of other pathophysiological mechanisms.

Despite the controversies from old studies, AER is being used in many hospitals today. The fact that we have found only one case published over the last 30 years presenting potential harm from AER would suggest a low risk of complications. In fact, there are numerous articles reporting successful rewarming using AER [47–49], and the general consensus among experts seems to be shifting [1, 2, 50–52].

This review does not mean to make a conclusion on whether it is safe to use AER in patients with AH. The aim is limited to describing published studies on severe haemodynamic or respiratory complications caused directly by the use of AER. One of the main findings of this review was the low number of articles, and the low quality of those that were found. Relevant cases may not be published because the authors do not believe that it will contribute anything new. Of course, it may also be because the use of AER is not as dangerous as previously perceived.

One specific subgroup may have good reason to be cautious with AER, chronic AH [26, 53]. Chronic hypothermia is most prevalent in the elderly population and can lead to a variety of pathophysiological changes. Shifts in fluid and electrolytes occur that are not immediately reversible and may require more controlled and steady correction [51, 54]. The cold diuresis will have had time to manifest for a prolonged period of time, and the patient may require fluid replacement therapy during rewarming.

#### Limitations

### Study quality

A systematic review is considered to be evidence-based science of high quality, summarizing data from multiple randomized controlled trials to synthesize all available knowledge on a specific subject. This review consists mostly of case reports, case series, and small clinical trials with low scientific value. Even though this is a systematic review of the available scientific literature, the quality of the scientific evidence is not as convincing as other systematic reviews. Therefore, the conclusions drawn from this review and the clinical implications must be cautious and reserved.

### Search strategies

We chose to exclude all studies involving invasive or extracorporeal techniques of rewarming in their title. However, it is obviously possible that some of the studies advocating the use of invasive warming techniques contain examples of why the use of external warming measures might be dangerous.

### Study selection process

During the initial screening for this study, 2200 articles were excluded. There is always a risk that some article titles did not immediately catch our attention and were falsely excluded. Also, 13 articles meeting our search criteria were unobtainable.

### Inclusion and exclusion criteria

We chose our inclusion criteria based on what kind of literature we thought would yield the most accurate answer to our research question. However, there may be, for example, animal or laboratory studies available that would have been of interest for this review.

## Conclusion

When conducting this review, we found no scientific evidence of acceptable quality to prove that the use of AER is dangerous for patients with AH in a prehospital setting.

We found several articles saying that AER is dangerous, but most of them do not cite references or provide evidence. It has become an undisputed fact without ever being proven to be true.

Several articles argue that the use of AER is safe and effective. These recommendations are not based on scientific evidence, but on the consensus of expert groups and commissions. This review can hopefully be a contribution to this growing body of evidence.

### Definitions

In the preparation phase of this systematic review, we found some inconsistencies in the literature regarding some key concepts [55]. Therefore, we deemed it necessary to clarify some of these terms in order to achieve a common understanding of the results found in this review.

#### Afterdrop

Afterdrop is exclusively a thermodynamic concept. It is defined as a continued decrease in the core temperature after extraction from the cold environment [56, 57]. The term afterdrop includes both the circulatory component (convective) and the direct conduction of heat in tissue (conductive] [27].

#### Rewarming syndrome

Rewarming syndrome is the combined result of all physiological and pathophysiological effects occurring during rewarming. The observed effects of rewarming syndrome consist of a rapid decrease in cardiac output and arterial blood pressure [58–60].

#### Rewarming shock

Rewarming shock is a state of circulatory shock following severe rewarming syndrome.

#### Circum-rescue collapse

Circum-rescue collapse is a pathophysiological phenomenon consisting of a sudden collapse of arterial blood pressure immediately prior to, during, or shortly after rescue from a hypothermic environment [56]. The term is frequently used in relation to extraction from water immersion.

#### Active warming

Active warming is when heat is supplied to the patient from an exogenous heat source [61].

#### Passive warming

Passive rewarming is when the patient is insulated from the environment, allowing endogenous heat production to increase the core temperature [48, 62].

#### External/non-invasive

External heating is when heat is applied to the skin, depending on convective or conductive heat transfer to increase the core temperature [61]. Examples of this modality is electrical blankets, forced air blankets, hot water immersion, heat packs, heated mattress, or hot water bottles [3, 47].

#### Extracorporeal rewarming

Invasive techniques in which the patient’s blood is circulated out of the body and rewarmed by a machine before being reinfused back into the patient’s own circulation [2, 63]. This can be accomplished by extracorporeal membrane oxygenation (ECMO), cardiopulmonary bypass, or haemodialysis.

#### Internal/invasive rewarming

Internal rewarming is when heat is applied directly to the patient’s core [51]. Examples of this is heated intravenous infusions; peritoneal-, gastric-, colonic-, or pleural lavage; or inhalation of heated air [51].

## Data Availability

Our research protocol, including detailed search strategies, is available online from the PROSPERO website: https://www.crd.york.ac.uk/PROSPERO/display_record.php?RecordID=107137

## Abbreviations

AH: Accidental hypothermia
AER: Active external rewarming
PER: Passive external rewarming
