# Correction to: Is prehospital use of active external warming dangerous for patients with accidental hypothermia: a systematic review

**DOI:** 10.1186/s13049-020-00802-0

**Published:** 2020-10-26

**Authors:** Sigurd Mydske, Øyvind Thomassen

**Affiliations:** 1 Department of Anaesthesia and Intensive Care, Haukeland University Hospital, Bergen, Norway; 2 Mountain Medicine Research Group, University of Bergen, Bergen, Norway; 3 Department of Research and Development, Norwegian Air Ambulance Foundation, Oslo, Norway

**Correction to: Scand J Trauma Resusc Emerg Med https://doi.org/10.1186/s13049-020-00773-2**

Following the publication of the original article [1], the authors became aware of two issues they would like to communicate to the readers:

1. In Fig. 1, second column, the number in the ‘Studies included’ box shows 9 instead of 8. The correct figure is included in this Correction.
2. In the ‘Study selection’ section, the symbol for the Kappa Coefficient shows up blank instead of (*κ*).

**Fig. 1:**
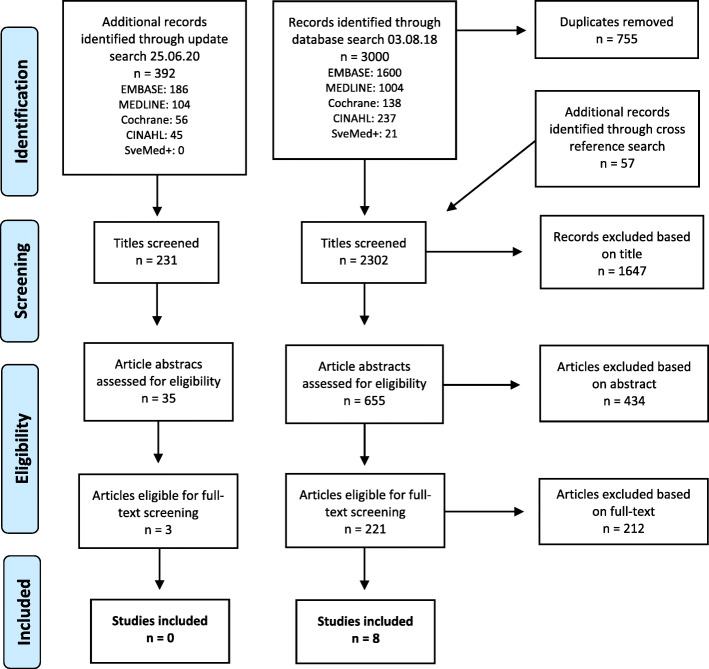
The PRISMA flow diagram showing the process of eligibility screening in our review

## References

[1] Mydske, Thomassen (2020). Is prehospital use of active external warming dangerous for patients with accidental hypothermia: a systematic review. Scand J Trauma Resusc Emerg Med.

